# Clinical evaluation of elderly people with chronic vestibular disorder

**DOI:** 10.1016/S1808-8694(15)30998-8

**Published:** 2015-10-19

**Authors:** Juliana Maria Gazzola, Fernando Freitas Ganança, Mayra Cristina Aratani, Monica Rodrigues Perracini, Maurício Malavasi Ganança

**Affiliations:** a Physical therapist. Gerontology specialist trained at UNIFESP - EPM. Graduate student (Master's degree) of Sciences at the UNIFESP - EPM Otorhinolaryngology and Head & Neck Surgery graduate course. FAPESP scholarship. Voluntary observing physical therapist at the Vestibular Rehabilitation Unit of the UNIFESP Otoneurology Department; b Physical therapist. Gerontology specialist trained at UNIFESP - EPM. Voluntary observing physical therapist at the Vestibular Rehabilitation Unit of the UNIFESP; c Physical therapist. Doctor in Rehabilitation Science graduated at UNIFESP - EPM. Coordinating Professor of the Physical Therapy Master's degree at the Sao Paulo City University. Voluntary observing physical therapist at the Vestibular Rehabilitation Unit of the UNIFESP; d Full Professor of Otorhinolaryngology at UNIFESP - EPM. Senior Researcher of the Graduate Program Stricto Sensu (Master's degree) on Neuromotor Rehabilitation Science at UNIBAN. Responsible for the Otoneurological Assessment Unit of the Otorhinolaryngology Sector at the Fleury Medical Diagnostic Center - Sao Paulo (SP); e Physician, ENT specialist, Doctor in Medicine trained at UNIFESP - EPM. Affiliated Professor of the Otoneurology Service at UNIFESP - EPM. Professor of the Neuromotor Rehabilitation Graduate Course at UNIBAN. Otoneurology Service at UNIFESP - EPM

**Keywords:** vestibular disease, elderly, dizziness

## Abstract

Dizziness is common among the elderly.

**Aim:**

To characterize social, demographic, clinical, functional and otoneurological data in elderly patients with chronic vestibular disorder.

**Method:**

A sequential study of 120 patients with chronic vestibular disorder. Simple descriptive analyses were undertaken.

**Results:**

Most of the patients were female (68.3%) with a mean age of 73.40±5.77 years. The average number of illnesses associated with the vestibular disorder was 3.83±1.84; the patients were taking on average 3.86±2.27 different medications. The most prevalent diagnosis on the vestibular exam was unilateral vestibular loss (29.8%) and the most prevalent etiology was metabolic vestibulopathy (40.0%) followed by benign paroxysmal positional vertigo (36.7%). Fifty-two patients (43.3%) had experienced dizziness for 5 years or more. Sixty-four patients (53.3%) had at least one fall in the last year and thirty-five (29.2%) had recurrent falls.

**Conclusions:**

Most of the sample included females with associated diseases, and using many different drugs. The most prevalent vestibular diseases were metabolic and vascular labyrinth conditions. Dizziness is a chronic symptom in elderly patients. The association of two vestibular diseases is common. Falls are prevalent in chronic dizzy elderly patients.

## INTRODUCTION

Vestibular dysfunction is a significant condition in the elderly, as age is directly proportional to the presence of multiple associated otoneurological symptoms such as dizziness, hearing loss, tinnitus, changes in body balance, gait disturbances and occasional falls, among others^1^.

Dizziness results from primary or secondary vestibular system disorders in approximately 85% of cases^2^.

Vertigo and other forms of dizziness originating in the vestibular system are frequent in the elderly. According to Ganança and Caovilla^3^ there are many citations on the prevalence of vertigo. It is present in 5 to 10% of the world population; it is the seventh most frequent complaint in women and the fourth most frequent complaint in men; it affects 47% of men and 61% of women over 70 years of age; it is the most common complaint in patients over 75 years of age; it is the second most common symptom in patients up to 65 years of age and the most common symptom in patients over 65 years of age; it is present 65% of persons aged 65 years and above, in 50% to 60% of the community dwelling elderly and in 81 to 91% of the elderly seen in geriatric outpatient clinics.

The most frequent otoneurological syndromes in the elderly are Benign Paroxysmal Positional Vertigo (BPPV), Ménière's Disease, Vascular Labyrinthopathies, Metabolic Labyrinthopathies, Presbivertigo/Presbiataxis/Presbitinnitus/Presbiacusis, Vestibular Neuritis, Labyrinthic Trauma, Ototoxicosis, Cervical Syndrome, Vestibular Migraine, Sudden Deafness, Immune Diseases, Vestibular Schwannoma, Vertebrobasilar Failure and Multiple Sclerosis^2^.

Tinetti et al.^4^ consider dizziness a geriatric syndrome, a multifactorial health condition that occurs due to the cumulative effect of multiple system deficits leading to increased vulnerability to circumstantial challenges in the elderly. These authors found that the activities and emotional states most frequently associated with dizziness are standing up, turning around and anxiety. They also noted that depression, balance disorders, previous acute myocardial infarction, postural hypotension, the number of medications taken, and hearing loss were clinical variables associated with an increased risk of dizziness. Furthermore, the probability of a report of dizziness was strongly associated with the number of predisposing variables.

A detailed clinical assessment looking at social, demographic, clinical, functional, and otoneurological data in the elderly with chronic vestibular disease could increase diagnostic precision and improve treatment.

The aim of this study was to characterize the social, demographic, clinical, functional, and otoneurological data of elderly patients with chronic vestibular dysfunction.

## METHODS

We conducted a cross-sectional descriptive study of a sample of male and female patients aged 65 years or more with chronic vestibular dysfunction, characterized by complaints of dizziness and/or imbalance and/or presyncope and/or other non-specific feelings of dizziness for at least the last three months. Patients were included sequentially from the Otoneurology Outpatient Clinic of the Paulista Medical School which belongs to the Sao Paulo Federal University (UNIFESP - EPM).

Exclusion criteria included elderly patients with crises of vertigo, patients with severely reduced visual and auditory acuity limiting completely the activities of daily living, even with corrective lenses and/or sound amplification devices, and patients that could not walk independently or that were on wheelchairs.

This study was approved by the UNIFESP - EPM Research Ethics Committee, protocol number 01215/05. All patients included in this study read the Information Letter and signed a free and informed consent form. This trial is part of a study funded by the Sao Paulo State Research Support Foundation (FAPESP), process number 03/10119-3.

Elderly patients initially underwent a clinical otoneurological assessment including a clinical history and an otorhinolaryngological physical exam, audiometry, imitanciometry and a vectoelectronystagmographic vestibular exam according to the Ganança et al.^2^, ^5^ guidelines.

Data were collected between April 2003 and November 2004.

The variables were classified into social and demographic data, clinical and functional data and otoneurological data.

Social and demographic data were gender, age, color, marital status, education level and housing conditions.

Clinical and functional data were the number of diseases, diseases as classified under the International Classification of Diseases^6^, the number of medications taken, drugs classified by the Anatomical Therapeutic Chemical (ATC) Classification Index^7^, activities, positions and symptoms related to the onset of dizziness, the use of walking aids and the occurrence of falls.

Otoneurological data included the syndromic and topographic diagnosis of the vestibular dysfunction, the number of associated vestibular conditions, the classification of vestibular dysfunction into the type of condition, the time from the onset of dizziness, the type, duration and periodicity of dizziness, the dizziness visual analog scale (VAS) and associated symptoms.

Data description analysis was done for statistical purposes. Analysis was done using the SAS System for Windows^8^ software.

## RESULTS

The study sample included 120 elderly patients with a diagnosis of chronic vestibular syndrome, monitored at the outpatient clinic. The average age was 73.4 years, the standard deviation (SD) was 5.77, and the maximum age was 89 years. Social and demographic data are shown on Table 1.

**Table 1:** Absolute and relative frequency of social and demographic data in 120 elderly patients with chronic vestibular dysfunction.

	Categories	Absolute Frequency (n)	Relative Frequency (%)
Gender	Male	38	31,7
	Female	82	68,3

Age group	65-69 years	36	30,0
	70-74 years	31	25,8
	75-79 years	37	30,8
	80 years or more	16	13,3

Color	White	84	70,0
	Yellow	4	3,3
	Black	14	11,7
	Mixed	18	15,0

Marital status	Married	66	55,0
	Not married	54	45,0

Education level	Illiterate / reads / writes	13	10,8
	Incomplete elementary	45	37,5
	Complete elementary	41	34,2
	Post-elementary	21	17,5

Housing arrangements	Alone	16	13,3
	With 1 generation	38	31,7
	With 2 or 3 generations	64	53,3
	Other	2	1,7

The average number of diseases per individual was 3.83 (SD=1.84), with a maximum of nine associated diseases; 29 patients (24.2%) had one or two diseases, 48 patients (40.0%) had three or four diseases and 43 patients (35.8%) had five or more diseases. Table 2 shows data on the prevalence of diseases classified according to the International Classification of Diseases^6^.

**Table 2:** Absolute and relative frequency of associated diseases in 120 elderly patients with chronic vestibular dysfunction.

	Absolute Frequency (n)	Relative Frequency (%)
Infectious and parasitic diseases	6	5,0
Neoplasms (tumors)	8	6,7
Diseases of the blood and hematopoietic organs and immune disorders	2	1,7
Nutritional endocrine and metabolic diseases	63	52,5
Mental and behavioral disorders	25	20,8
Diseases of the nervous system	12	10,0
Diseases of the eye and annexes	35	29,2
Diseases of the circulatory system	88	73,3
Diseases of the respiratory system	12	10,0
Diseases of the digestive system	14	11,7
Diseases of the skin and subcutaneous tissue	2	1,7
Diseases of the osteomuscular system and connective tissue	76	63,3
Diseases of the genitourinary system	15	12,5

The average number of medications taken by patients was 3.86 (SD=2.27), with a maximum use of 10 medications. Table 3 shows the number of medications used and Table 4 show the classification of medications according to the ATC^7^.

**Table 3:** Absolute and relative frequency of the number of medications used by 120 elderly patients with chronic vestibular dysfunction.

	Categories	Absolute Frequency(n)	Relative Frequency (%)
Number of medications	Non-user	4	3,3
	1 or 2 medications	35	29,2
	3 or 4 medications	37	30,8
	5 or more medications	44	36,7

**Table 4:** Absolute and relative frequency of medications used by 120 elderly patients with chronic vestibular dysfunction, according to the Anatomical Therapeutic Chemical Classification Index.

	Absolute Frequency (n)	Relative Frequency (%)
Use of otoneurological drugs	76	63,3
Use of cardiovascular system drugs	81	67,5
Use of GI tract and metabolic system drugs	46	38,3
Use of drugs for the nervous system	36	30,0
Use of hematological system drugs	44	36,7
Use of muscle skeletal system drugs	16	13,3
Use of respiratory system drugs	4	3,3
Use of systemic hormone preparation drugs	12	10,0
Use of ophthalmologic drugs	10	8,3
Use of other drugs	6	5,0

Twelve elderly patients (10.0%) used walking aids.

64 elderly patients (53.3%) had at least one fall during the last year. Of these 35 (54.6%) reported recurrent falls. Most patients reported fear of falls (72.5%) and the tendency to fall (79.2%).

Topographic diagnosis of vestibular dysfunction by the vestibular exam was done in 105 of 120 elderly patients. Fifteen patients had not undertaken the vestibular exam until the time of assessment. The vestibular exam disclosed the following diagnoses, in order of prevalence: Vestibular Deficiency Syndrome in 39.0% of cases (15.2% in the right ear, 14.3% in the left ear and 9.5% in both ears), Irritative Vestibular Syndrome in 32,4% of cases (4.8% in the right ear, 2.9% in the left ear, 12.4% in both ears and 12.4% which were undetermined), Normal in 20.0% of patients, Central Vestibular Syndrome in 7.6% of cases, and Mixed Vestibular Syndrome in 1.0% of cases.

The prevalence of the number of vestibular conditions was classified as follows: one disease (45.0%), two diseases (38.3%) and three or more diseases (6.7%). Vestibular diseases diagnosed in the sample of 120 patients are shown on Table 5 in decreasing order of prevalence.

**Table 5:** Absolute and relative frequency of vestibular conditions in 120 elderly patients with chronic vestibular dysfunction.

	Absolute Frequency (n)	Relative Frequency (%)
Metabolic Labyrinthopathy	48	40,0
Benign Positional Paroxysmal Vertigo	44	36,7
Vascular Labyrinthopathy	27	22,5
Idiopathic Cochleovestibular Syndrome	22	18,3
Vertebrobasilar Failure	19	15,8
Ménière's Disease	15	12,5
Presbivertigo/Presbiataxis/Presbitinnitus/Presbiacusis	13	10,8
Cervical Syndrome	7	5,8
Vestibular Migraine	3	2,5
Vestibular Schwannoma	3	2,5
Labyrinthic Trauma	3	2,5
Ototoxicosis	1	0,8
Immune Disease	1	0,8
Vestibular Neuritis	1	0,8

VAS quantified reported self-perception of the intensity of dizziness in elderly patients with chronic vestibular dysfunction revealed an average of 6.62 points (SD=2.45), with zero as a minimum score and ten as a maximum score for some patients.

The time from onset of dizziness prevalence was 3 to 6 months (10.8% of cases), 7 to 11 months (8.3%), 1 to 2 years (25.0%), 3 to 4 years (12.5%) and over 5 years (43.3%).

Characterization of dizziness according to type, duration and periodicity is shown on Table 6.

**Table 6:** Absolute and relative frequency of the type, duration and periodicity of dizziness in 120 elderly patients with chronic vestibular dysfunction.

	Categories	Absolute Frequency (n)	Relative Frequency (%)
Type of dizziness	Rotating dizziness	8	6,7
	Non-rotating dizziness	30	25,0
	Both	82	68,3
Type of rotating dizziness	Subjective	49	40,8
	Objective	35	29,2
	Both	6	5,0
	No report	30	25,0

Duration of dizziness	Days	14	11,7
	Hours	23	19,2
	Minutes	45	37,5
	Seconds	38	31,7

Periodicity of dizziness	Sporadic	15	12,5
	Monthly	17	14,2
	Weekly	33	27,5
	Daily	55	45,8

Activities, positions and predisposing symptoms of dizziness are shown in decreasing order on Table 7.

**Table 7:** Absolute and relative frequency of predisposing activities, positions and symptoms of dizziness in 120 elderly patients with chronic vestibular dysfunction.

	Absolute Frequency (n)	Relative Frequency (%)
Turning the head	81	67,5
Head in a specific position	77	64,2
Arising from decubitus	70	58,3
Walking	69	57,5
During exercise	62	51,7
Arising from the seated position	60	50,0
Turning the body from a sitting or standing position	54	45,0
When anxious	53	44,2
Changing position in bed	40	33,3
Lying down on one side	22	18,3
Sitting still	7	5,8

Associated auditory symptoms included hypersensitivity to sounds in 90 elderly patients (75.0%), followed by tinnitus (70.8%), auditory deficits (60.0%), and a feeling of aural pressure/fullness (54.2%). The prevalence of reported neurovegetative symptoms was sweating/pallor/tachycardia (43.3%), nausea (43.3%), vomiting (17.5%) and presyncope (40.8%). We highlight the importance of psychological symptoms in the elderly with vestibular disease, including anxiety (74.2%), memory and concentration deficits (68.3%) and fear (40.0%). Other common signs and symptoms encountered in the sample were oscillopsia (59.2%), insomnia (48.3%) and headache (42.5%).

## DISCUSSION

The main social and demographic characteristics of our elderly patient sample were similar to those of other studies of elderly patients monitored in otoneurology outpatient clinics with a complaint of dizziness^3^, ^4^, ^5^, ^6^, ^7^, ^8^, ^9^, and similar to the profile of elderly persons dwelling in Brazilian urban regions according to Ramos et al.^14^

Ramos et al.^14^ found a relatively young population, with 58.0% of persons aged below 70 years and an average age of 69 years. However, in our sample of elderly patients with vestibular disease we found an older population, similar to Ebel's^9^ study. The average age was relatively high (73.4 years), similar to reports by Ebel^9^ (74.3 years), Gushikem^10^ (72.0 years), Cavalli^11^ (70.5 years) and Simoceli et al.^13^ (72.3 years).

Most of the elderly patients in our sample were women, which is similar to the findings of Ramos et al.^14^ in community dwelling elderly persons (60.0%) and also similar to elderly patients with vestibular disease in outpatient clinics studied by Ebel^9^ (68.3%), Gushikem^10^ (67.6%) and Medeiros^12^ (64.5%).

Campos^15^ reported that dizziness is more frequent in women in a 2:1 ratio. The association of vestibular disease, hormone dysfunction, and metabolic disorders in women and the fact that women tend to seek medical help more frequently than men could justify this prevalence^16^.

Over half of our elderly patient sample lived in multigeneration families, which is also similar to the findings of Ramos et al.^14^ in community dwelling elderly persons (59.0%). However, we found no studies showing the housing arrangements of elderly patients with vestibular diseases to compare with our study.

Our elderly patient sample contained a high proportion of elderly persons with low education levels; only 17.5% had post-elementary schooling. This is similar to post-elementary schooling percentages found by Ramos et al.^14^ (18.0%) and Medeiros^12^ (11.9%).

Ramos et al.'s^14^ population study revealed that most of the elderly persons (80.0%) had at least one chronic disease, and that a small percentage of this group (10.0%) had at least five diseases. In our elderly patient sample we found a prevalence of five or more chronic diseases, other than the vestibular syndrome, in 35.8% of patients. Our elderly patient sample included voluntary participants from an otoneurology outpatient clinic, which could mean a biased population with increased health problems compared to community dwelling elderly persons in general.

The main vestibular disease etiologies found in elderly patients in our study were metabolic and vascular causes, similar to observations by Mangabeira Albernaz^17^ and Ganança et al.^18^ in patients with dizziness, regardless of age. These numbers were also similar to studies of elderly patients with a complaint of dizziness monitored in outpatient settings^9^, ^10^.

Metabolic labyrinth disease, particularly in the elderly, may arise from metabolic disorders such as hyperlipidemia, hyper or hypoglycemia, hyperinsulinism or insulinopenia, uremia, hyper or hypothyroidism, and ovarian hormonal alterations, among others. It may also be caused by dietary errors, which may lead to labyrinth disorders and/or act as significant aggravating factors. Metabolic disorders may promote vestibular symptoms varying from mild instability to clinical pictures similar to Ménière's disease^5^, ^15^, ^19^, ^20^. Bittar et al.^21^ stated that in glucose metabolism, both hypoglycemia and hyperglycemia have a major influence on the inner ear, altering its usual function. Insulinemic alterations in the long term may be responsible for accelerating the development of atherosclerotic lesions in diabetes mellitus patients^21^, one of the most prevalent diseases in the elderly^22^.

According to Ganança et al.^23^, arterial hyper or hypotension, heart failure, myocardial infarction, arrhythmias, hypersensitivity of carotid sinus reflexes, aortic stenosis and atherosclerosis are the main circulatory disorders causing peripheral and/or central affections of the auditory and/or vestibular systems. Auditory and vestibular lesions tend to be attributed to ischemic disorders resulting from decreased blood perfusion, lower oxygen supply and/or intravascular obstacles (embolism and atherosclerosis).

An important point is that some of the vestibular diseases found in our elderly patient sample could also have been classified as having a vascular origin. This is due to compromise of blood flow to the peripheral and/or central vestibular systems, such as in the case of diabetes mellitus and hyperlipidemias - classified in this study as having a metabolic origin - and BPPV and vertebrobasilar failure. As such, vascular vestibular diseases would become the most common vestibular conditions in this age group.

We also found a higher prevalence of osteomuscular and connective tissue disorders in our elderly patient sample compared to findings by Ebel^9^ (38.3%) and Gushikem^10^ (41.2%). Various aging conditions of the osteoarticular system such as physiological osteopenia, cartilaginous aging, sarcopenia and reduction in nerve conduction velocity are cited. Pain and muscle and skeletal disorders are the most frequent complaint presented by elderly patients, as the incidence of many rheumatological diseases, including osteoporosis, osteoarthritis, rheumatic polymyalgia and others, increases with age^24^.

Results of the vestibular exam in this study was similar to those found by Caovilla et al.^25^, in which 88.7% were peripheral vestibular syndromes, 11.0% were central vestibular syndromes, and 03.% were mixed syndromes. In 43 patients with vestibular dysfunction and ages ranging from 14 to 88 years, Whitney et al.^26^ found that 79.0% were peripheral vestibular syndromes, 7.0% were central vestibular syndromes and 14.0% were multisensory conditions.

Ganança et al.^27^ and Sloane and Baloh^28^ reported that BPPV is the most common vestibular affection in the elderly. In our study the most prevalent affection was metabolic labyrinthopathy. The diagnostic hypothesis of metabolically originated vestibular disease was considered when patients presented alterations related to carbohydrate and/or lipid metabolism, altered uric acid values or altered thyroid and pancreatic hormone levels in the absence of other vestibular clinical pictures. As metabolic disorders are common in elderly patients, idiopathic conditions may have been inadequately classified as being metabolically, given the diagnostic exclusion process made to diagnose idiopathic affections by discarding other vestibular diseases.

Less common vestibular disorders in our elderly patient sample were ototoxicosis, immune diseases, vestibular neuritis, vestibular migraine, vestibular schawnnoma, labyrinthic trauma and cervical syndrome. These were similar findings to those of Caovilla et al.^25^ in a retrospective study of one thousand new consecutive patients from a private clinic, aged between 21 and 80 years, medically and otoneurologically diagnosed as having vestibular disorder.

Although there was a small prevalence of the cervical syndrome, it is important to emphasize that, upper cervical column osteoarticular pathophysiological mechanisms such as osteophytes, atlanto-occipital instability, and cervical root or articular facet inflammation or irritation may cause dizziness due to alterations in cervical proprioception^29^.

The prevalence of vestibular affections in this study was 45.0% for one disease and was 55.0% for two or more diseases, similar to findings by Simoceli et al.^13^, in which 49.0% of elderly patients had a single etiology and 51.0% had two or more etiologies for imbalance, demonstrating the increased involvement of the vestibular system in this age group.

In our study most of the elderly patients used medication. Users of five or more medications were 36.7% of our sample, and the average was 3.86 medications per patient, similar to findings in elderly patients in a study by Gushikem^10^. Values found in our study were higher compared to those disclosed by Garcia^30^, who found that 72.0% of community dwelling elderly persons used medical medications, with an average 2.05 medications per patient. This is probably because elderly patients with vestibular disorders, having more health problems, require more medication.

The most common medical drugs used by our elderly patient sample were medications for the cardiovascular system, for GI tract conditions, for metabolic disorders, for hematological diseases and for nervous system affections. According to Garcia^30^, users of five or more medications were 11.2% of patients. The most common group of medications were those for the cardiovascular system, followed by medications for the GI tract, for metabolic disorders, for the nervous system and the hematological system. No published reports were found in literature reporting the most commonly used types of medications in elderly patients with vestibular diseases.

The incidence of falls in our elderly patient sample differed from population studies. Campbell et al.,^31^ Tinetti et al.^32^, and Perracini and Ramos^33^ showed a 30.0% to 35.0% prevalence of falls during the last year in community dwelling elderly persons. Our elderly patient sample with vestibular disorders had a higher prevalence (53.3%), similar to a study by Medeiros^12^ (59.2%).

Recurring falls in our study were found in 29.2% of patients, different from a study by Perracini and Ramos^33^ in metropolitan Sao Paulo, where the prevalence was 11.0%.

Vestibular originated dizziness may facilitate falls and recurrent falls. According to Herdman et al.^34^, vestibular dysfunction limits posture control, including stability and body alignment. These authors found a statistically significant higher number of falls in patients with bilateral vestibular dysfunction aged between 65 and 74 years compared to community dwelling elderly persons in the same age group.

There was a predominance of associated rotating and non-rotating dizziness in our patients, different from Caovilla et al.'s^25^ study in which rotating dizziness predominated.

Our elderly patient sample had long-term dizziness, similar to the study by Medeiros^12^, in which 77.6% patients reported dizziness for over one year. Sloane and Baloh^28^ found that dizziness had an average duration of 36.2 months, demonstrating the chronic nature of dizziness in the elderly population, and the difficulty of vestibular compensation in this age group.

Predisposing symptoms for dizziness reported in this study were turning the head, keeping the head in a specific position, arising from decubitus, walking, and anxiety, similar to findings by Tinetti et al.^4^, who described the most common positions or activities as being arising from decubitus, turning the head, turning the body, standing from a sitting position, and anxiety. These head movements and/or positions strongly stimulate the vestibular system and frequently produce vertigo and other forms of dizziness such as in BPPV, which is so prevalent in elderly patients.

Anxiety is a psychical symptom, different from motor activities related to head movements and balance control that predispose to dizziness. Psychic symptoms may be caused by psychic insecurity secondary to physical insecurity generated by balance disturbances^35^.

The prevalence of associated symptoms was higher in elderly patients in our study compared to the sample in Caovilla et al.^25^, study, in which the prevalence of associated symptoms in patients with vestibular diseases in general was 24.9% for tinnitus, 20.2% for hypoacusis, 18.3% for headaches, 14.1% for depression, 12.6% for memory disturbances, 12.6% for hypersensitivity to sounds sons, 12.5% for nausea, 10.3% for anxiety, 10.1% for presyncope, 10.1% for feeling of aural pressure/fullness, 9.5% for fear, 7.7% for sweating/pallor/tachycardia and 5.1% for vomiting. Our data agrees with Gushikem's^10^ findings, which reveal an association between dizziness and tinnitus in 79.4% of cases, hypoacusis in 55.9%, sensitivity to loud sounds in 47.1%, neurovegetative disturbances in 55.9% and syncope in 8.8% of cases in elderly patients with vestibular diseases. These findings corroborate the fact that elderly patients with vestibular diseases tend to have a concomitant presentation of other systems related to vestibular functioning, among which the auditory system.

## CONCLUSION

The sample of elderly patients with chronic vestibular dysfunction assessed in this study included mostly women and a high average age, with associated diseases and polypharmacotherapy. The conditions most frequently associated with vestibular dysfunction were circulatory, nutritional endocrine, metabolic, and osteomuscular diseases. The most frequently used medications were cardiovascular and otoneurological drugs. The most frequent vestibular diseases and topography were metabolic and vascular labyrinthopathy and unilateral deficient peripheral syndrome. Dizziness was a chronic or long-term condition. The association between two vestibular disorders was common. The association between rotating and non-rotating dizziness was frequent. Falls and other symptoms were prevalent in this population.

## References

[1] Ganança MM, Caovilla HH, Ganança MM (1998). Vertigem tem cura?.

[2] Ganança MM, Caovilla HH, Munhoz MSL, Silva MLG, Frazza MM, Ganança MM, Caovilla HH, Munhoz MSL, Silva MLG (1999). Equilibriometria Clínica. Série Otoneurologia.

[3] Ganança MM, Caovilla HH, Ganança MM, Vieira RM, Caovilla HH (1998). Princípios de Otoneurologia. Série Distúrbios de Comunicação Humana.

[4] Tinetti ME, Williams CS, Gill TM (2000). Dizziness among older adults: a possible geriatric syndrome. Ann Intern Med.

[5] Ganança MM, Caovilla HH, Munhoz MSL, Silva MLG, Ganança FF, Ganança CF (1999). A vertigem explicada. RBM Rev Bras Med.

[6] Organização Mundial de Saúde (2000). CID-10. Classificação estatística internacional de doenças e problemas relacionados à saúde.

[7] World Health Organization (1992). Anatomical Therapeutic Chemical (ATC) Classification Index. WHO Collaborating Center for Drug Statistics Methodology.

[8] The SAS Statistical Analysis System: system for Windows [computer program]. Version 6.12. Cary (NC): SAS; 1996

[9] Ebel SJ (1994). Prevalência de Sintomas e Sinais Otoneurológicos em Pacientes Idosos com Queixa de Tonturas [Dissertação].

[10] Gushikem P (2001). Avaliação Otoneurológica em Idosos com Tontura [Dissertação].

[11] Cavalli SS (2003). Avaliação da qualidade de vida em Idosos com tontura que apresentaram e não apresentaram quedas. [Dissertação].

[12] Medeiros RFR (2003). Estudo da Berg Balance Scale em idosos vestibulopatas. [Dissertação].

[13] Simoceli L, Bittar RMS, Bottino MA, Bento RF (2003). Diagnostic approach of balance in the elderly: preliminary results. Rev bras otorrinolaringol.

[14] Ramos LR, Rosa TEC, Oliveira ZM, Medina MCG, Santos FRG (1993). Perfil do idoso em área metropolitana na região sudeste do Brasil: resultados de inquérito domiciliar. Rev Saúde Pública.

[15] Campos CAH, Ganança MM (1998). Vertigem tem cura?.

[16] Pedalini MEB, Bittar RSM, Formigoni LG, Cruz OLS, Bento RF, Miniti A (1999). Reabilitação vestibular como tratamento da tontura: experiência com 116 casos. Arquivos da Fundação Otorrinolaringologia.

[17] Mangabeira Albernaz PL (1982). Aspectos otoneurológicos na velhice. Acta AWHO.

[18] Ganança MM, Caovilla HH, Munhoz MSL, Silva MLG, Settanni FAP, Ganança FF, Munhoz MSL, Ganança MM, Caovilla HH, Silva MLG (2001). Casos Clínicos Otoneurológicos Típicos e Atípicos.

[19] Freitas MR, Weckx LLM (1998). Como diagnosticar e tratar labirintopatias. RBM Rev Bras Med.

[20] Bittar RSM, Bottino MA, Zerati FE, Moraes CLO, Cunha AU, Bento RF (2003). Prevalência das alterações metabólicas em pacientes portadores de queixas vestibulares. Rev Bras Otorrinolaringol.

[21] Bittar RSM, Sanchez TG, Santoro PP, Medeiros IRT (1998). O metabolismo da glicose e o ouvido interno. Arquivos da Fundação Otorrinolaringologia.

[22] Nasri F, Freitas EV, Py L, Neri AL, Cançado FAX, Gorzoni ML, Rocha SM (2002). Tratado de Geriatria e Gerontologia.

[23] Ganança MM, Caovilla HH, Munhoz MSL, Silva MLG, Settanni FAP, Silva MLG, Munhoz MSL, Ganança MM, Caovilla HH (2000). Quadros clínicos otoneurológicos mais comuns. Série Otoneurológica vol. 3.

[24] Sader CS, Rossi E, Freitas EV, Py L, Neri AL, Cançado FAX, Gorzoni ML, Rocha SM (2002). Tratado de Geriatria e Gerontologia.

[25] Caovilla HH, Ganança MM, Munhoz MSL, Silva MLG, Frazza MM (1997). O valor da nistagmografia computadorizada. Rev Bras Med Otorrinolaringol.

[26] Whitney S, Wrisley D, Furman J (2003). Concurrent validity of the Berg Balance Scale and the Dynamic Gait Index in people with vestibular dysfunction. Physiother Res Int.

[27] Ganança MM, Caovilla HH, Munhoz MSL, Silva MLG (1999). Alterações da audição e do equilíbrio corporal no idoso. RBM rev bras med.

[28] Sloane PD, Baloh RW (1989). Persistent dizziness in geriatric patients. J Am Geriatr Soc.

[29] Clendaniel RA, Herdman SJ (2002). Reabilitação Vestibular.

[30] Garcia JT (2000). Padrão de uso de medicamentos em idosos residentes na comunidade urbana: a importância de polimedicação (projeto EPIDOSO) [Dissertação].

[31] Campbell AJ, Reinken J, Allan BC, Martinez GS (1981). Falls in old age: a study of frequency and related clinical factors. Age ageing.

[32] Tinetti ME, Speechley M, Ginter SF (1988). Risk factors for falls among elderly persons living in the community. N Engl J Med.

[33] Perracini MR, Ramos LR (2002). Fatores associados a quedas em uma coorte de idosos residentes na comunidade. Rev Saúde Pública.

[34] Herdman SJ, Blatt P, Schubert MC, Tusa RJ (2000). Falls in patients with vestibular deficits. Am J Otol.

[35] Caovilla HH, Ganança MM, Munhoz MSL, Silva MLG, Frazza MM (1998). Dicas sobre vestibulopatias periféricas e centrais. Rev Bras Med Otorrinolaringol.

